# Family Members' Perceptions of Their Psychological Responses One Year Following Pediatric Intensive Care Unit (PICU) Hospitalization: Qualitative Findings From the Caring Intensively Study

**DOI:** 10.3389/fped.2021.724155

**Published:** 2021-09-07

**Authors:** Janet E. Rennick, Alyssa M. Knox, Stephanie C. Treherne, Karen Dryden-Palmer, Robyn Stremler, Christine T. Chambers, Lyndsey McRae, Michelle Ho, Dale M. Stack, Geoffrey Dougherty, Hailey Fudge, Marsha Campbell-Yeo

**Affiliations:** ^1^Department of Nursing, The Montreal Children's Hospital, McGill University Health Centre (MUHC), Montreal, QC, Canada; ^2^Ingram School of Nursing, Faculty of Medicine and Health Sciences, McGill University, Montreal, QC, Canada; ^3^Department of Pediatrics, Faculty of Medicine and Health Sciences, McGill University, Montreal, QC, Canada; ^4^Child Health and Human Development, Research Institute of the MUHC, Montreal, QC, Canada; ^5^Department of Critical Care, Hospital for Sick Children, Toronto, ON, Canada; ^6^Child Health Evaluative Sciences, Research Institute, Hospital for Sick Children, Toronto, ON, Canada; ^7^Lawrence S. Bloomberg Faculty of Nursing, University of Toronto, Toronto, ON, Canada; ^8^Department of Psychology and Neuroscience and Department of Pediatrics, Dalhousie University, Halifax, NS, Canada; ^9^Department of Neurosciences and Trauma, Hospital for Sick Children, Toronto, ON, Canada; ^10^Division of Paediatric Medicine, Complex Care Program, Hospital for Sick Children, Toronto, ON, Canada; ^11^Department of Psychology and Centre for Research in Human Development, Concordia University, Montreal, QC, Canada; ^12^Department of Epidemiology and Biostatistics, Faculty of Medicine and Health Sciences, McGill University, Montreal, QC, Canada; ^13^School of Nursing, Faculty of Health, Dalhousie University, Halifax, NS, Canada; ^14^Department of Nursing and Department of Pediatrics, IWK Health, Halifax, NS, Canada

**Keywords:** pediatric intensive care, psychological outcomes, longitudinal follow-up, children, family, pediatrics, post-intensive care syndrome

## Abstract

**Introduction:** PICU hospitalization can have a profound impact on child survivors and their families. There is limited research on children's long-term recovery within the context of the family following critical illness. This study aimed to explore children's and parents' perceptions of long-term psychological and behavioral responses within the context of the family one year following PICU hospitalization.

**Materials and Methods:** Caring Intensively is a mixed methods multi-site prospective cohort study that aims to examine children's psychological and behavioral responses over a 3-year period following PICU hospitalization. In this study, part of the qualitative arm of Caring Intensively, an interpretive descriptive design was used to explore children's recovery one year post-discharge. Purposive sampling was used to select 17 families, including 16 mothers, 6 fathers, and 9 children. Semi-structured, audio-recorded interviews were conducted. Data were analyzed iteratively using the constant comparison method.

**Results:** Families described efforts to readapt to routine life and find a new normal following PICU hospitalization. Finding a New Normal consisted of four major themes: (1) Processing PICU Reminders and Memories, (2) Changing Perceptions of Health and Illness, (3) We Are Not the Same, and (4) Altered Relationships. Participants described significant emotional and behavioral changes during the year following discharge. The psychological impact of individual family members' experiences led to changes in their sense of self, which affected family dynamics. PICU memories and reminders impacted participants' perceptions of childhood health and illness and resulted in increased vigilance. Parents and siblings demonstrated increased concern for the child survivor's health, and the experience of long absences and new or altered caregiving roles resulted in changes in relationships and family dynamics.

**Conclusion:** PICU hospitalization impacted the psychological well-being of all family members as they sought to re-establish a sense of normalcy one year following discharge. Parent and child experiences and responses were closely interconnected. Findings highlight the importance of increased follow-up care aimed at supporting the family's psychological recovery.

## Introduction

Pediatric intensive care is designed to save lives and improve child health outcomes; and yet, while mortality rates have steadily declined over the last four decades, the number of pediatric intensive care unit (PICU) survivors with moderate to severe morbidities has increased dramatically (1–3). The experience of critical illness can have profound and lasting impacts on children's and family members' psychological health and well-being (4–9). The Integrative Trajectory Model of Pediatric Medical Traumatic Stress (PMTS) suggests that children and their families can experience traumatic stress responses as a result of a child's serious illness, injury, and/or exposure to painful, invasive or frightening treatment procedures, and that those responses can continue long after the acute event (10, 11). In fact, post-traumatic stress symptoms are experienced by 35–62% of PICU survivors (5, 12–14), and 5–34% of children are diagnosed with PTSD (5, 13, 15). Up to 20% of survivors are at higher risk of general psychiatric disorders (15). As many as 20–30% of children who survive PICU hospitalization demonstrate lower health-related quality of life (HRQoL) than healthy peers at 3 months post-PICU, and HRQoL remains significantly worse at 12 months in those who are also experiencing post-traumatic stress symptoms (5, 8, 16). Children also report changes in self-esteem and sense of self, increased anxiety, sleep disturbances, medical fears, and changes in friendships and social identity (14, 17, 18). Moreover, children describe their critical illness experiences as significant and challenging, and report a sense of disruption in their lives as they cope with exposure to death and dying during PICU hospitalization, and deal with changes in identity and social relationships after discharge (18, 19).

The impact of a child's critical illness on family members can also be profound. Post Intensive Care Syndrome in pediatrics (PICS-p) is a new framework that conceptualizes the morbidities experienced by child survivors, highlighting the impact of a child's critical illness on family members, and the interdependency between child and family outcomes (6). This interdependency is alarming as up to 84% of parents report post-traumatic stress symptoms, and between 10 and 21% are diagnosed with post-traumatic stress disorder following their child's PICU hospitalization (8). Parents report high levels of anxiety (up to 60%) and depression (up to 50%), describe distressing memories, and acknowledge being newly overprotective of their children (4, 9, 13, 20). Negative impacts on the mental health and well-being of siblings have been reported by parents, but there have not been studies directly exploring siblings' self-reported experience when a brother or sister is hospitalized in the PICU (4, 21). Impacts on family members are not only concerning in and of themselves, but may influence the child survivor's ongoing well-being (6, 9). There is limited research on child recovery within the context of the family following critical illness. The importance of the relationship between child and parent well-being was illustrated in Atkins et al.'s (22) model of the post-PICU journey for families. They subsequently explored the strategies families employed to navigate their post-PICU experience, and identified four major themes which informed a recovery path framework: parents' and children's changed perceptions of themselves and their relationships, journeying to a new sense of normality, the need to develop a narrative, and the experience of positive growth during recovery (23). These findings emphasize the interdependence between family members and the centrality of the family unit in influencing the child's trajectory of recovery.

Research conducted to-date has largely focused on the child's experience or individual family member's psychological responses following PICU hospitalization. Little is known about how family members' responses impact one another, and there is a dearth of literature on children's recovery trajectories within the context of the family (9). In order to prevent or minimize psychological sequelae following a child's critical illness, healthcare professionals need to better understand family members' perceptions of their psychological responses following PICU hospitalization. The objective of this study was to explore children's and parents' perceptions of their psychological and behavioral responses to PICU hospitalization within the context of the family one year following discharge.

## Materials and Methods

Caring Intensively is a mixed methods multi-site prospective cohort study that uses a concurrent triangulation design (24) to examine children's psychological and behavioral responses over a 3-year period following PICU hospitalization (7). The current study focused on interview data collected at one year following PICU hospitalization from families enrolled in the qualitative arm of Caring Intensively.

Children hospitalized in the PICUs of three Canadian university-affiliated pediatric hospitals and their parents were recruited in the Caring Intensively study, which received ethics approval from the Research Ethics Boards at each participating hospital site. Parents provided written, informed consent for their children and themselves to participate, and children provided written or verbal assent (depending upon provincial law) to participate. Participants' names were removed from the data files and replaced by study numbers, and all data files were password protected to ensure confidentiality.

### Study Design

An interpretive descriptive approach was used to explore the contextual and unique nature of participants' experiences following PICU hospitalization, with attention paid to the psychological and behavioral impacts of PICU hospitalization on the recovering child (25, 26).

### Participants

The Caring Intensively study included English and French-speaking children aged 3–12 years who were admitted to the PICU for a minimum of 24 h, and their parents. Children who had undergone a previous PICU admission, or who experienced a severe brain injury that prevented them from responding to standardized measures (used in the quantitative arm) were excluded (7). Purposive sampling was used to select a sample representative of the overall study cohort. We selectively included families of children representing a variety of ages, PICU lengths of stay, diagnostic categories and hospital sites. Families were contacted ~11 months post-PICU discharge to schedule a year-1 follow-up interview. We aimed to conduct interviews between 12 and 15 months post-PICU discharge.

### Data Collection

Semi-structured audiorecorded interviews took place in the preferred language of participants one year following PICU discharge. Interviews were conducted in the family home, a location of their choosing, or over the phone. Interview questions were used to elicit participants' perceptions of their children's psychological and behavioral responses following PICU hospitalization, while probing questions promoted the elaboration of recollections and the clarification of their responses (Table 1). Interviewer field notes were collected to extend participants' descriptions, capture their non-verbal responses, and provide context to any interruptions and distractions that occurred during the interview. Member checks were conducted with study participants during data collection to validate data interpretation and help guide subsequent interviews.

**Table 1:** Interview questions.

Child interview questions^*^	**Probe(s)**
Do you remember being in the intensive care unit? Can you tell me what it was like?	Were there good things about being there? Not so good things?
When you came home from the hospital, did anything seem different from before?	How did your sisters/brothers/parents respond when you came home? Have things gone back to the way they were before you were in the hospital?
Do you talk about your time in the intensive care unit with anyone?	What do you talk about? With who?
How did your friends react when you came home from the hospital?	How did that make you feel?
What was it like when you went back to school?	Were there things you were worried about when you went back to school? How did your friends react when you went back to school? How are things going now?
**Parent interview questions**	**Probe(s)**
What was it like for you to be in the intensive care unit?	What are your memories of that time? What stands out for you? What was it like when you came home from the hospital?
What do you think it was like for [child] in the intensive care unit?	Do you and your child ever talk about his/her hospitalization?
How has your family routine changed since [child] returned home?	
Have you noticed any changes in your child since coming home?	When did [described changes] take place? Do you have any concerns about them? How has your child responded to [described changes]?
Did you notice any changes in the behavior of your other children following [child's] hospitalization?^**^	When did these changes take place? Do you have concerns about [the changes]? Has [sibling] brought up any concerns about [child survivor's] health with you?
Some parents have shared that the time following their child's PICU stay was difficult for them. Can you tell us how you have been feeling since [child's] hospitalization?	Is there anyone you talk to about your feelings/your concerns? How has your partner been feeling? (If partner not present)
Has [child] had any medical follow up since he/she was in the PICU?	How did [child] respond?

* *Phrasing of child interview questions was adapted by the interviewer according to the child's age*.

** *Question regarding sibling responses was added after 3 interviews when it became apparent, based on parents' responses, that this was an important area to continue to explore*.

### Data Analysis

Family demographic and hospital baseline information were analyzed using descriptive statistics. Audio-recorded interviews were transcribed verbatim and combined with field notes. Interview data were analyzed using the constant comparison method (27, 28). Three members of the study team (JR, AK, ST) read the transcripts independently and conducted line-by-line coding to describe key components of the data. NVivo software (29) was used to store, index, retrieve, sort and code the data, helping to enhance study rigor (30, 31).

Data collection and analysis was approached iteratively, and it became apparent after the first few interviews that the data we had set out to collect differed from the data that we were, in fact, collecting. As parents drew links between their children's psychological and behavioral responses to PICU hospitalization and their own responses, emerging connections were validated and explored in subsequent interviews. As the interviews proceeded it became apparent that multiple family members experienced profound emotional and behavioral changes during the year following PICU hospitalization. This resulted in significant tension in the data analysis process as it became increasingly clear that child and parent responses could not be separated, nor should they be. A decision was made by the team to hear stories of recovery within the context of the family's experience, and to add probes to questions that had previously unintentionally dichotomized parent and child experiences (Table 1). This responsive approach to the data is consistent with the underlying tenets of interpretive description and is necessary for recognizing the complexity of the phenomena under study (25). Study findings reflect what family members felt was important for health care professionals to know about their responses following PICU hospitalization.

As interviews and data analysis progressed, new codes were organized into broad categories and compared within and across interviews in consultation with a fourth member of the study team (KDP) to identify commonalities and variations. Recurring data across categories were extracted and condensed and final themes identified (32, 33). Data saturation was considered to have been achieved when new information produced little or no change in data categories or themes and no new items were emerging (34, 35).

The trustworthiness of the study findings was established through multiple strategies. Credibility was enhanced by including families of children requiring PICU hospitalization for a variety of reasons, reflecting the diversity of those enrolled in the larger cohort. Member checks were conducted with study participants during data collection to validate data interpretation and help guide subsequent interviews. Triangulation of multiple data sources, including field notes and interview transcripts, and having multiple team members analyse and interpret the data contributed to the credibility and confirmability of the study findings. Finally, the dependability and transferability of the findings was enhanced by establishing and maintaining a clear audit trail outlining the complex decision-making process that took place as the data were analyzed.

## Results

Twenty-six families were approached to be interviewed, and 17 families completed an interview between 12 and 15 months post-discharge. Seven families were unable to schedule an interview within the required time period; two families did not respond after repeated attempts to confirm scheduled interviews. Of the 17 families interviewed, participants included 9 children (6 boys and 3 girls), 16 mothers, and 5 fathers (Table 2). While siblings had not been invited to participate in the interviews as this was not part of the original study objectives, parents described sibling responses in 16 of the 17 interviews (one child did not have any siblings at the time of hospitalization).

**Table 2:** Family demographics and hospital baseline characteristics (*n* = 17)^*^.

	***n* (%)**	$\bar{x}$ **(SD)**
**Households**		
One-parent household	2 (11.8)	–
Two-parent household	15 (88.2)	–
Household members	–	4.5 (1)
Parent age (years)	–	36 (4.9)
Child age at enrolment (years)	–	6.4 (2.6)
**Parent highest level of education**		
High school	3 (17.6)	–
College	3 (17.6)	–
University	11 (64.8)	–
Parent employed (vs. not employed)	15 (88.2)	–
**Family member(s) participating in interview**		
Mother and child	5 (29.4)	–
Both parents and child	4 (23.5)	–
Mother only	6 (35.3)	–
Father only	1 (5.9)	–
Both parents only	1 (5.9)	–
**Child PICU primary diagnostic category**		
Respiratory	5 (29.4)	–
Cardiology	2 (11.7)	–
Neurology	6 (35.3)	–
Trauma	1 (5.9)	–
Oncology	1 (5.9)	–
Infectious disease	1 (5.9)	–
Nephrology	1 (5.9)	–
Child PICU length of stay (days)	–	5.2 (5)
Child total hospital LOS (days)	–	25 (47.5)
Invasive procedure score (36)	–	149.1 (225.9)
Pediatric risk of mortality score (PRISM III) (37)	–	5.25 (7.2)
**Recruitment site**		
MCH	8 (47.1)	–
SickKids	6 (35.3)	–
IWK	3 (17.6)	–

* *Family demographics were collected at the time of Caring Intensively study enrollment and were completed by one parent in each family (n = 17)*.

Twelve interviews were conducted at home, two interviews with families living >150 km from the hospital took place by telephone, and three interviews were conducted outside the home as preferred by participants. All of the child interviews took place in the home. In-person interviews were attended by two study team members; one conducted the interviews and one collected observational field notes. The average interview length was 1 h (30–110 min).

### Finding a New Normal Following PICU Hospitalization

**Figure 1:**
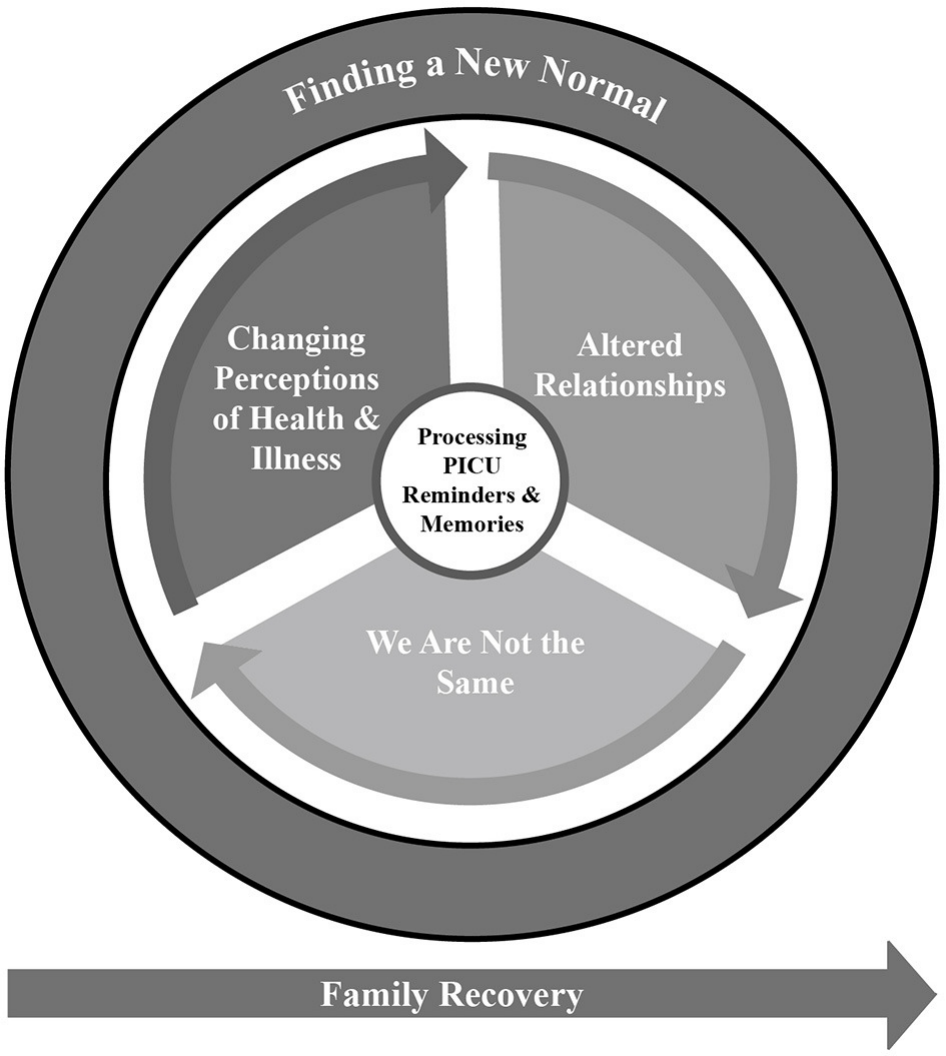
Establishing family normalcy following PICU hospitalization.

Families described efforts to readapt to routine life and find a new normal following PICU hospitalization. Finding a New Normal was identified as the overarching theme, and it consisted of four major themes: (1) Processing PICU Reminders and Memories, (2) Changing Perceptions of Health and Illness, (3) We Are Not the Same, and (4) Altered Relationships (Figure 1). Participants recounted how their memories and reminders of the child's PICU hospitalization continued to have a significant emotional impact on daily life. The child's acute illness resulted in disrupted family routines, including prolonged parental absences and changes in family relationships. Many participants felt they were not the same person as before the PICU. They reported changed perceptions of the child survivor's health and safety, including heightened vigilance on the part of parents and siblings toward the child and, for some child survivors, fear of becoming sick again or returning to hospital.

#### Processing PICU Reminders and Memories

Participants, especially parents, remembered the PICU stay vividly. Family separation presented a significant challenge during hospitalization, resulting in feelings of abandonment for parents alone at the PICU bedside and siblings at home missing their parents. Children and families kept mementos and marked anniversaries to make meaning by commemorating the PICU experience. This theme included three subthemes: Like it just happened yesterday, “You weren't there with me,” and Commemorating the PICU experience.

##### Like It Just Happened Yesterday

Parents described their child's PICU stay in vivid detail and were often emotional as they reflected on their experiences. One mother stated, “It's like it happened just yesterday. I think every parent who goes through any pain with their child [will] have it with them forever.“ She later said, “I'd have moments on my own where I'd cry or something would remind me…. I still cry, like if I think of something, I'll cry about it” (Family 6: F6). Several children either spontaneously talked about their PICU experiences with their families, or reacted when exposed to specific reminders of that experience (F15, F16, F2, F3, F7, F8, F9). Reminders included music and toys from the hospital, physical surroundings or objects similar to, or found in the hospital (e.g., stethoscopes, lab coats), or seeing the hospital itself. These reminders were often emotionally charged. One mother explained, “if [we see a] long hallway or anything that looks hospital-like, [my child says] ‘I don't wanna stay. We're not sleeping here, are we?”’ (F9). Parents also reported experiencing flashbacks and emotional reactions to reminders of their child's PICU stay: “When we came back [home], it was OK. I was taking care of him. It's later, when you start remembering, because you have these images coming to your mind” (F2). A few children had significant reactions when returning to hospital for follow-up care: “We couldn't calm him down enough [to start the test]. So we tried for an hour to calm him down, nothing worked” (F8).

##### You Weren't There With Me

Families faced multiple and sometimes prolonged separations. Often one parent remained at the bedside throughout the hospital stay while the other remained at home. Sometimes the parent in hospital attempted to protect their spouse from the emotional strain of receiving difficult or uncertain information about their child's condition: “[I was] alone at that time. I didn't even call home… until I got the right answer from the ICU, I didn't call my husband” (F4). Others felt abandoned by partners or other family members who were not there. One mother explained:

“…when they're telling you [child's diagnosis]… you don't wanna be the only one, on your own, listening to that. I was very angry with [my husband] at that time because I felt I was alone. His sister went in with me, but it's not the same. You don't have your partner” (F6).

Another mother explained how separation from her partner continued to have an emotional impact months after hospitalization.

“Sometimes at night… I start crying and screaming [at my husband] for nothing. I said “What happened? Why just me? I saw everything” …Sometimes I feel I'm still there. Whenever I'm not happy, I go back… I'm suffering again and I start to scream” (F4).

Siblings at home were separated from the parent at the hospital (often their primary caregiver) for extended periods of time. One mother reflected: “I think it's the siblings who suffer, perhaps more. You take [the hospitalized child] out of the family synergy; then mommy too. We concentrate on [the hospitalized child] a lot, but I think a lot of parents have to say that we forget about the others” (F16).

##### Commemorating the PICU Experience

Children and families recognized and marked the PICU experience through anniversary celebrations and keeping mementos from the hospital such as toys and medical devices. One child explained the meaning of her stuffed toys from the hospital: “They are really important, they make me feel good. They remind me of the hospital and what was said to me after at my ballet recital [about being proud of my recovery]” (F16). Another child showed the interviewer how he still played with the inhaler spacer he received in hospital. In one family, both the child and a sibling shared their PICU experience by making presentations about it at school (F16). Another child made a presentation about her experience and wrote a poem about her thoughts before having surgery, which she still revisited at times (F11). Some families marked the anniversary of their child's PICU discharge: one family went out for dinner to celebrate, another collected hospital donations from their community, and another family became involved with the Children's Wish Foundation. These different approaches to commemorating the PICU experience helped families mark the passage of time and recognize the importance of their experience.

#### Changes in Perceptions of Health and Illness

The child's critical illness challenged parents' preexisting perceptions of “normal” childhood health and illness and revised the notion that childhood illness is temporary and minor. Some parents initially had difficulty understanding that their child was critically ill, and ultimately redefined their conceptualizations of illness and health following their experience of critical illness and PICU hospitalization. Parents and siblings perceived the child as more vulnerable, as did some of the child survivors themselves. This new perception of vulnerability lead to anxiety and increased vigilance: “We're all obviously more cautious now. We can see how things can escalate” (F7).

##### Heightened Vigilance

Most parents remembered keeping extremely close watch over their child in the PICU. They described feelings of not wanting to leave their child's side and the need to witness and monitor the child's responses to care. Parents described themselves as “overprotective” or “on guard,” and they remained that way at home following discharge. One mother explained, “Every time I hear her cough, I'm like, ‘What is that? Why are you coughing?’ She's like ‘It's just a cough.’ No, [for me] there's no such thing” (F7). One mother reported that her husband worried about interactions between the recovered child and her sibling “[Her father] wouldn't let [her brother] hug her… He was afraid that [brother] was gonna hurt her, squeeze her too hard” (F11). Several parents watched their child's breathing or checked their child's temperature at night, and one family bought a stethoscope to listen to their child's lungs. Parents worried about having necessary medications available at all times: “We have a rescue drug now … I worry when we go out that I've forgotten it” (F5). Indeed, one year later most families reported remaining vigilant, on the lookout for any small change in the child's health. One mother explained, “even my kids will do it, ‘Mom, [child] is not well, should we get the temperature?”’ (F6). One mother whose child had become immunocompromised experienced changes in her own friendships and social group: “You don't care if you lose a few friends…. If you have diarrhea, you're sneezing, you have a cold or any type of virus or whatever, please don't come [to my house]. I really don't need you that much” (F3).

##### The Vulnerable Child

Families responded differently when the child experienced any injury or illness, exemplified by one family's reaction to the child's fall at school: “I got a panic phone call from my younger son that ‘[Child] fell at school, [child] is crying, daddy is crying’, and he was crying. I'm like ‘Okay, I'm hanging up, I'm on my way”’ (F11). Another parent whose child was at risk of his illness recurring explained, “I'm scared I could lose him any time. That's my biggest problem” (F3). Parents described being quick to worry or react to a change in their child's health status, and second guessed their reactions: “You try not to overreact but it's hard” (F15). One parent explained, “now they get a cold and I'm like on edge” (F9). Parents were more likely to seek a second opinion, or take their child to hospital. Siblings, too, worried more: “[Her brother] has often said, ‘Does my sister have to go back into the hospital?”’ (F11).

Some child survivors felt vulnerable: one girl talked about her surgical scar, stating she didn't want her body to be touched that way again (F16). Another child became more concerned about her asthma: “If she starts having an asthma episode, she comes to me right away. It became more serious to her” (F7). One child was described as “scared… she's a little bit nervous about getting sick again” (F17). Parents expressed concerns about recurrence of the illness itself, and the possibility of other unrelated injuries or illnesses. One mother expressed fear for her child's health, and the knowledge that this fear was unfounded:

“For me it's like, I'm still there. I know he's a normal child now. Everybody knows that, except me. When he's playing with his brothers and I'm sitting with the rest of the family, my heart is over there…. If it's another kid, “You have a bobo, no problem.” If he says he has one, my god it's not normal” (F4).

#### We Are Not the Same

Most parents felt that recovering emotionally and psychologically from their child's PICU hospitalization was a long process that was still ongoing one year post-discharge. They described how focused their families had been on the child who was in the PICU, and an emotional adjustment to life after discharge that went beyond practical changes to include psychological and emotional changes affecting all family members. This was expressed in terms of reestablishing oneself as a parent, and changes in the child that rendered them different than they were before PICU hospitalization.

##### Re-establishing Myself as a Parent

After PICU discharge, parents continued to feel they had to set aside their own needs to focus on caring for their child. Several parents' professional lives were significantly disrupted by their child's PICU stay and they took leaves of absence, left jobs, or put educational goals on hold during the hospital stay and to care for their child after discharge (F2, F4, F6, F15). One family initially sought support in adapting to their changed life post PICU from a psychologist, but did not continue as they felt the psychologist lacked understanding of their particular situation: “We were under the impression that she was treating us like a psychologist in general and not within our context… it's the fact of always being confronted with… disability, the fact that he can't do things like everyone else, so constantly, in such a regular and structured way [that stresses me]” (F8). These parents described not having space in their new post-PICU life to attend to their own or their child survivor's emotional needs. A mother who experienced lightheadedness, but whose health was found to be normal, attributed her symptoms to intense anxiety about her child's health (F10). Some parents felt they had changed significantly as a result of their child's hospitalization. One mother stated, “I was an easygoing mom before… it's hard. I miss the relaxed me, a lot” (F9). Another parent explained that at one year post-discharge, she was simply not yet herself: “I need more time to be a normal mother” (F4).

##### Different Than Before: Children's Changes

Many children were reported to have experienced changes in their health status after their PICU stay. Some recovered to pre-hospitalization physical levels slowly after surgical interventions, while others experienced significant and sustained changes in their level of ability, such as a new hemiplegia or cognitive changes. Changes in body image were concerning for some children: “She doesn't like her right pinky finger. She says it looks different from the other finger, which it does. And I said different is good” (F6). Some children struggled with performing activities of daily living. One mother explained: “He's having difficulty learning, he can't eat by himself, he can't go to the bathroom [alone]. It is hard. He's really trying.” (F3) While some families were able to take the child's current abilities and health status in stride, others found it more challenging as they adjusted, for example, to caring for a new medical device such as a child's gastrostomy tube, pacemaker, or peripherally inserted central line.

Changes in the child survivor's behavior and emotions were identified by parents. In describing their child at one year post-discharge, one couple said: “He's not the same person. He changed a lot. He became mature, he's not acting like a kid. … But [we're] starting to see a change; he's becoming more and more familiar” (F4). Several parents felt their children were generally “more emotional” since hospitalization (F4, F9, F15, F16). Others noted specific changes in sleep habits (not being able to sleep alone, wetting the bed, nightmares), emotional expression (anger, anxiety), and social behavior (hitting siblings, playing alone more often, being more shy or less confident than before). One child described changes in his behavior: “When I came home, I was shy about talking with my parents. I didn't talk a lot… I was quite shy when I talked” (F4).

One child developed physical symptoms (i.e., cough and stomach aches) after discharge that were determined to be psychosomatic in origin: “He can throw up…he gets nervous and plays with his clothes… it's anxiety. Before the hospital he was pretty fearless” (F9). Two other families felt their children were using physical complaints to get out of activities they did not want to engage in (F8, F18). One mother described a series of changes in her daughter's social behavior after a brief PICU stay: “She became really chatty when we came home. And that lasted… at least a month, and then she got clingy… [for] a couple of months. And then now she seems pretty normal to me” (F13). Three families described changed and problematic behavior at home (e.g., hitting siblings) while noting their children's teachers reported no issues at school (F4, F16, F15).

Some parents, particularly of children who experienced long PICU stays, described significant emotional and behavioral changes in siblings that often came to light at school. These took the form of behaviors like withdrawing from social situations or falling uncharacteristically behind on homework (F2, F3, F4, F11). For some siblings, these issues resolved once the parent was back in the home full time. For other families, sibling disruptions continued and in one case required ongoing psychological support. One parent described a sibling's newly developed fear of visitors to the home: “She is very afraid of people that enter the house. I think it's because she has this memory that when someone comes to the house, it means her parents are going to leave” (F8).

#### Altered Relationships

PICU hospitalization constituted a significant disruption of family life, and some families experienced sustained relationship changes. This included parents' relationships with the recovered child, the child's relationships at school, relationships between parents, and relationships between the child and their siblings. In this area four subthemes were identified: Altered parent-child relationships, Fitting in at school, I want to be special too, and Trying to buffer against negative memories.

##### Altered Parent-Child Relationships

Returning home meant re-establishing a disrupted family routine. All children needed some level of follow-up care—from simple rest, to medication and follow-up appointments, to extensive rehabilitation in the home and at outpatient facilities that, for some, lasted months after hospital discharge. Within the context of these changes, some parents felt they had been able to re-establish a normal family routine fairly quickly—while others, particularly those who endured longer PICU stays, found it harder. One mother explained, “When she came home I felt quite vulnerable and I forgot how to have a home environment because I [had been] away so long, myself” (F6). Parents described multiple changes in their relationships with their children. Some parents became more focused on the child survivor: “I love my other children but now my priority is to be close to [child]” (F10). The mother of a child with cancer in remission explained, “You just want to spend every moment, every second with your child” (F3).

In some families, parents and siblings were described as “babying” the child (F10, F6). In turn, one child survivor worried about how being present at the bedside impacted her mother's job, and how visiting her in the PICU impacted her father's emotional well-being (F11). Siblings were reported to have assumed some of their parents' previous responsibilities, including conveying information to the school regarding the child's health status (F2), accepting new roles/tasks at home, and helping monitor the child survivor's health. When asked what he would do if his sister fell down the stairs due to changes in balance, one younger sibling felt prepared: “I would call daddy and grandma and 911” (F11).

##### Fitting in at School

Returning to school was a significant milestone for school aged children. Five children were not yet in elementary school at the time of their PICU stay. Among those who were in school, some children were absent only a week or two (F1, F7, F9, F17), some missed 1 month (F11, F16), and others were absent for more than 1 month (F2, F3, F4, F6, F18). Some children returned to school with supportive accommodations such as a modified school day, a classroom aide, or modified transit. Four children changed schools because of cognitive and physical changes, developmental transitions, or a family move, adding additional stressors to the return to school period. Most families, including those with lengthy PICU stays, reported that their child's return to school or their initial entrance to school or preschool in the year after the PICU stay was smooth.

Some children received support from their peers after returning to school. For example, one child's friends helped her regain her sense of confidence, while another child's friend provided physical assistance on her return to school. One child reported making new friends who had also had surgeries and finding significant support in those peer connections. Some children faced a changed social experience at school because of new health-related limitations (F3, F6, F11). One mother noted, “It's scary for other children to see him; he has difficulty adjusting, or other kids have difficulty adjusting to him… so, it's hard” (F3). Another mother explained: “It was upsetting for her that she couldn't play, and she couldn't swim. The neighbors all have pools so they wouldn't invite her over. So that was a little stressful on her” (F11). One father of a preschool-aged child worried about potential future bullying his child might face due to physical changes after hospitalization (F8).

##### “I Want to Be Special Too”

Parents perceived their child's PICU experience as emotionally challenging for siblings. The absence of a parent, often the primary caregiver, was considered a significant stressor (F1, F3, F15, F16). Several siblings struggled with feelings of jealousy as they perceived the child in the PICU got to be more “special” or to receive more attention and alone time with their parents. One mother explained “[Her sister] was torn, she said ‘I don't want to have heart surgery either, but I'd like to be special too”’ (F16). Siblings across all developmental stages acted out, from a preschool-aged child testing boundaries, to a young school-aged child having difficulty sharing toys after a long period of solo play at home, to an older elementary-aged child who exhibited behavioral difficulties over a significant period of time at school and was ultimately expelled. The mother of this older sibling qualified his behavior as a call for help: “They don't know how to express themselves besides getting bad attention, causing trouble” (F3). Child survivors with siblings who felt left out or jealous had hospital lengths of stay that varied from a single week to a period of months.

##### Trying to Buffer Difficult Memories

Fundamental to pediatric critical care is the role of the professional care team to protect the child from harm and minimize difficult care experiences. Many children reported positive PICU memories linked to interventions intended to create positive and supported experiences. They recalled toys gifted in hospital, therapeutic clowns, and positive play experiences. One mother explained, “Even when she first came home… she was bragging about [her PICU stay] to her older sister and older brother, and it just wasn't a negative thing completely” (F13). Three children had positive memories of staff members and wanted to visit them when they returned to the hospital for follow-up appointments (F3, F11, F15). One mother reflected: “I think this will become one of his childhood memories, but I wouldn't say that it is a dark memory” (F1).

Parents reported actively working to protect their child following the PICU experience by attempting to hide their own feelings or reactions: “I think [child] could sense my nervousness for a while, but I learned to hide it better” (F9). In one case, parents observed similar behavior in the siblings: “[her brothers] rarely tell you how they're feeling. They try to hide it, I think so we don't get upset by seeing them upset” (F12). Some parents encouraged their children to develop positive memories and associations. One father explained, “I think despite all the hard times she went through, a lot of those really hard memories are kind of fading away… And I think hopefully we've been reinforcing the good memories” (F15).

## Discussion

The experience of a PICU admission continued to have a significant impact on all participants at one year post-discharge. Parents remembered the PICU hospitalization like it had just happened yesterday, and related vivid and emotionally charged accounts of their own and their children's experiences. The psychological impact of individual family members' experiences led to changes in their sense of self which, in turn, impacted family dynamics. PICU memories and reminders impacted participants' perceptions of childhood health and illness and resulted in increased vigilance. Parents and siblings demonstrated increased concern for the child survivor's health, and the experience of long absences and new or altered caregiving roles resulted in changes in relationships and family dynamics (Figure 1).

As they attempted to re-establish a sense of normalcy post-PICU hospitalization, families progressed along different trajectories of recovery that appeared similar to some of those identified in the Integrative Trajectory Model of Pediatric Medical Traumatic Stress (10, 11). For some families, significant self-reported psychological challenges persisted at one year following their PICU stay, while for others those challenges seemed to have been resolved or were gradually resolving. This is also consistent with the Post-Intensive Care Syndrome in pediatrics (PICS-p) framework (6), which proposes that recovery trajectories in children and families will vary following discharge. The Caring Intensively study, which the current study is a part of, is following families over a 3-year period post-PICU discharge and will shed light on how these trajectories unfold over time (7).

Child and parent responses to PICU hospitalization have generally been studied independently (4); however, our findings suggest they are closely interrelated and must be conceptualized, explored and addressed together. We found the impacts of experiences, memories, and sequelae were shared by parents, the child survivor, and siblings (as reported by parents). This lends support to the PICS-p framework (6) that highlights the family as an interdependent unit in which all members must be considered together when examining children's responses. In our study, this was reflected in the family's reactions to changes in the child's health. Parents, siblings, and child survivors noticed how their emotional responses to this stressful life event impacted one another, and they attempted to protect each other by managing their emotional expressions. The ongoing impacts of family members' individual psychological challenges, and their attempts to hide those same challenges from one another, have yet to be fully understood. In particular, heightened vigilance and changed family relationships may have ongoing impacts on children's and other family members' mental health, potentially contributing to the evolution of post-PICU psychological sequelae over time.

Our results expand upon those of other studies that have sought to understand families' experiences following PICU hospitalization. Atkins et al. (23) interviewed children aged 5–16 years and their parents, between 8 and 18 months following the child's PICU hospitalization. Their findings included participants' reports of personal change, and of the importance of striving for normalcy. Terp and Sjostrom-Strand (38) interviewed the parents of children aged 0–5 years, 2 years following PICU hospitalization, and identified the presence of vivid memories and the significant impact of family separations. Children were reported to experience sleep disturbances and anxiety which gradually resolved over time, and ongoing anxiety associated with going to the hospital; sequelae reported by participants in our study, which included children aged 3–12 years. We found that psychological sequelae were experienced not only by children, but also parents and siblings. Indeed, the whole family went to the PICU. In addition, we found that all family members' perceptions of health and illness changed after the PICU experience, and that this was an important driver of change in relationships. In a systematic review synthesizing thematic data from three qualitative studies of PICU survivors up to one year after discharge, Manning et al. (17) highlighted that child survivors struggled to remember and talk about their PICU experiences, and their parents' narratives influenced how they thought and felt about their hospitalizations. These findings resonate with our participants' attempts to manage the impacts of their feelings, fears and emotions on family members, reinforcing the importance of the interdependence of this phenomenon and that family members' psychological impacts are both shaping and shaped by one another.

The impact of PICU hospitalization on the child's siblings is long recognized but under-explored (21). In particular, there is an absence of sibling self-report data regarding either the PICU hospitalization period or its aftermath. Parents interviewed by Terp and Sjostrom-Strand (38) observed increased anxiety and overprotectiveness in siblings. While our study's original focus was on child survivors' and not on siblings' responses to the child's PICU hospitalization, the extent to which parents felt their child's PICU stay had impacted siblings was striking. Siblings felt left out during and following PICU hospitalization, and parents felt the separations experienced by siblings were themselves traumatic. Siblings were vigilant about the child survivor's health and assumed additional responsibilities in the family. Our findings reinforce the importance of further research exploring siblings' experiences during and after PICU hospitalization, and the role they play in family trajectories of recovery.

Families in our study faced challenges attaining psychosocial support after leaving the PICU. These challenges included a lack of protected time for therapy given the child survivor's care needs, and perceptions that their specific situation was poorly understood by the mental health professionals with whom they had contact. There is the potential for critical care specialists to address this gap. Previous studies have explored the feasibility and acceptability of PICU follow-up clinics, which would be well-positioned to understand and address family coping with both psychological distress and medical concerns post-PICU (39–44). Obtaining support within the context of a PICU follow-up clinic would provide families with access to multidisciplinary teams, including mental health care professionals who understand their particular context and care needs, prepare families to understand the changes they are experiencing, help to normalize the recovery process, and facilitate the mobilization of family resources. Presenting mental health services as a routine part of PICU follow-up might make parents more likely to seek support for themselves; parents in our study were likely to focus on their child's needs above their own, and many noted that in spite of significant distress they had elected not to seek professional support, but rather attempted to cope on their own.

## Limitations

While we aimed to include children who had been in the PICU in these interviews, the decision of whether or not to include them was left up to their parents. Children from 9 of 17 families participated, with a mean age of 7 years. Interview scripts were developed for parents and children, however child interview strategies such as incorporating play for younger children were not included and may have limited their interest in participating. In addition, children may have been reluctant to share their experiences in the presence of their parents.

While the sample was purposively selected to be representative of families included in the Caring Intensively study at baseline, those who consented to participate and were able to accommodate the interview could have been different from those who did not participate. Families who were not able to schedule an interview might have been experiencing more challenging situations or, conversely, might have moved on from the PICU experience and been less interested in discussing that time in their lives.

## Conclusion

PICU hospitalization impacted the psychological well-being of all family members as they sought to re-establish a sense of normalcy one year following discharge. Parent and child experiences were closely interconnected and PICU hospitalization remained a vivid and emotionally charged memory. Family members' perceptions of health and illness changed, leading to a new perception of the child survivor as vulnerable. The presence of continued parent and sibling vigilance around the child survivor's health, and significant alterations in family relationships, were acknowledged sources of concern that may lead to long-term impacts on children's and family members' psychological well-being following PICU hospitalization. This highlights the importance of increased follow-up care aimed at supporting psychological recovery. The Caring Intensively study will continue to examine psychological and behavioral responses and how trajectories of recovery unfold up to 3 years after PICU hospitalization, and will inform and refine our approach to this important phenomenon (7).

## Data Availability Statement

The dataset generated and analyzed during the current study are not publicly available given that complete interview data are confidential. Representative quotes are included in this published article. Requests regarding the dataset should be directed to janet.rennick@muhc.mcgill.ca.

## Ethics Statement

This study was reviewed and approved by the McGill University Health Center Research Ethics Board (REB Approval #12-350-PED), the SickKids Research Ethics Board (REB Approval #1000041389), and the IWK Health Center Research Ethics Board (REB Approval #1014431). Written informed consent to participate in this study was provided by the participants' legal guardian/next of kin.

## Author Contributions

JR is the Principal Investigator and contributed to study design, conduct, data analysis and interpretation, and the writing of the manuscript. AK contributed to data analysis and interpretation and the writing of the manuscript. ST conducted family interviews and contributed to data analysis and interpretation and the writing of the manuscript. KD-P contributed to study design, conducted family interviews, contributed to data analysis and interpretation, and the writing of the manuscript. RS and CC oversaw the conduct of the study as site-based PIs, contributed to study design, and edited the manuscript. LM and MH conducted family interviews and edited the manuscript. DS and GD contributed to study design and edited the manuscript. HF contributed to writing the manuscript. MC-Y oversaw the conduct of the study as site-based PI, contributed to study design, data interpretation, and edited the manuscript. All authors read and approved the final manuscript.

## Funding

This study was funded by the Canadian Institutes of Health Research (CIHR: 123287). JR was a Fonds de recherche du Québec – Santé (FRQ-S) Clinical Research Scholar. JR and GD are members of the Research Institute of the McGill University Health Center which is funded by the FRQ-S. MC-Y was supported through a Canadian Institutes of Health Research (CIHR) New Investigator Award. RS holds the Lawrence S. Bloomberg Professorship in Child and Family Health.

## Conflict of Interest

The authors declare that the research was conducted in the absence of any commercial or financial relationships that could be construed as a potential conflict of interest.

## Publisher's Note

All claims expressed in this article are solely those of the authors and do not necessarily represent those of their affiliated organizations, or those of the publisher, the editors and the reviewers. Any product that may be evaluated in this article, or claim that may be made by its manufacturer, is not guaranteed or endorsed by the publisher.
